# Hexosamine-Induced TGF-*β* Signaling and Osteogenic Differentiation of Dental Pulp Stem Cells Are Dependent on N-Acetylglucosaminyltransferase V

**DOI:** 10.1155/2015/924397

**Published:** 2015-10-25

**Authors:** Yi-Jane Chen, Chung-Chen Yao, Chien-Hsun Huang, Hao-Hueng Chang, Tai-Horng Young

**Affiliations:** ^1^Department of Dentistry, National Taiwan University Hospital and School of Dentistry, National Taiwan University, Taipei City 10048, Taiwan; ^2^Institute of Biomedical Engineering, College of Medicine and College of Engineering, National Taiwan University, Taipei City 10048, Taiwan

## Abstract

Glycans of cell surface glycoproteins are involved in the regulation of cell migration, growth, and differentiation. N-acetyl-glucosaminyltransferase V (GnT-V) transfers N-acetyl-d-glucosamine to form *β*1,6-branched N-glycans, thus playing a crucial role in the biosynthesis of glycoproteins. This study reveals the distinct expression of GnT-V in STRO-1 and CD-146 double-positive dental pulp stem cells (DPSCs). Furthermore, we investigated three types of hexosamines and their N-acetyl derivatives for possible effects on the osteogenic differentiation potential of DPSCs. Our results showed that exogenous d-glucosamine (GlcN), N-acetyl-d-glucosamine (GlcNAc), d-mannosamine (ManN), and acetyl-d-mannosamine (ManNAc) promoted DPSCs' early osteogenic differentiation in the absence of osteogenic supplements, but d-galactosamine (GalN) or N-acetyl-galactosamine (GalNAc) did not. Effects include the increased level of TGF-*β* receptor type I, activation of TGF-*β* signaling, and increased mRNA expression of osteogenic differentiation marker genes. The hexosamine-treated DPSCs showed an increased mineralized matrix deposition in the presence of osteogenic supplements. Moreover, the level of TGF-*β* receptor type I and early osteogenic differentiation were abolished in the DPSCs transfected with siRNA for GnT-V knockdown. These results suggest that GnT-V plays a critical role in the hexosamine-induced activation of TGF-*β* signaling and subsequent osteogenic differentiation of DPSCs.

## 1. Introduction

Dental pulp stem cells (DPSCs) are clonogenic, rapidly proliferative, and capable of forming woven bone in vitro and compact bone in vivo [1–4]. It is important to explore the factors that trigger the osteogenic differentiation of DPSCs for their potential use in bone tissue engineering. Glucosamine (GlcN) is a prominent precursor in the biosynthetic process of glycosylated proteins. Uridine diphosphate N-acetyl glucosamine (UDP-GlcNAc) acts as a glycosyl donor in glycosylation reactions [5]. Oligosaccharides on cell surface glycoproteins play an important role in many cellular events, such as transmembrane signaling, cell adhesion, proliferation, and differentiation [6–9].

N- and O-linked glycosylations are two common cotranslational and posttranslational modification reactions [8, 10]. Glycoprotein receptors have evolved with low (1 or 2 N-glycan sites) or high (8~16 N-glycan sites) numbers of N-linked glycans [6]. Branches of N-glycans in the ER and Golgi apparatus are modified by the sequential action of N-acetylglucosaminyltransferases I (GnT-I), II (GnT-II), IV (GnT-IV), and V (GnT-V), which are, respectively, encoded by Mgat1, Mgat2, Mgat4a/b, and Mgat5 [8, 11]. GnT-III and GnT-V are the two major glycosyltransferases in the biosynthesis process of N-linked glycoproteins. GnT-III transfers UDP-GlcNAc to form bisecting branched N-glycans [12]. GnT-V transfers UDP-GlcNAc to form branching and elongated N-linked glycans, thus playing a critical role in sugar chain elongation. The most common type of O-linked glycosylation is the addition of O-linked *β*-N-acetylglucosamine (O-GlcNAc) [10]. O-linked N-acetylglucosamine transferase (OGT) catalyzes the addition of O-GlcNAc residue from the donor UDP-GlcNAc to the carrier protein. Conversely, O-GlcNAc can be removed from the carrier protein by O-linked N-acetylglucosaminidase (O-GlcNAcase) [13].

Metabolic flux through the hexosamine pathway was reported to regulate UDP-GlcNAc levels in the Golgi and the glycoprotein levels on the cell surface, for example, epithelial growth factor receptor (EGFr) and other growth factor receptors in high-number N-glycans as well as transforming growth factor *β* receptor (TGF-*β*r) in low-number N-glycans [6]. The change in hexosamine concentration induces a switch-like response in regulating the level of TGF-*β*r, which is one of the important receptors in the transduction of TGF-*β*/Smad signaling pathway. The elevation of intracellular UDP-GlcNAc increases N-glycan branching and thus may enhance retention of growth factor receptors at the cell surface via association with galectin-3, subsequently leading to an increase in signaling.

Previous studies have reported that exogenous GlcN promotes tissue regeneration of dental pulp wounds [14] and induces early osteogenic differentiation of DPSCs by modulating the level of transforming growth factor-*β* receptor (TGF-*β*r) type I [15]. It is not clear whether other hexosamines and their N-acetyl derivatives can regulate the osteogenic differentiation of DPSCs. In this study, we report the predominant expression of GnT-V in DPSCs at both the transcriptional and translational levels. Our study further aimed to evaluate possible effects of three types of hexosamines and their N-acetyl derivatives on the osteogenic differentiation potential of DPSCs and to investigate whether GnT-V is involved in the effects of hexosamine derivatives.

## 2. Materials and Methods

### 2.1. Reagents and Chemicals

Dulbecco's modified Eagle's medium (DMEM), penicillin G-streptomycin sulfate, phosphate-buffered solution (PBS), trypsin-EDTA, and fetal bovine serum (FBS) were purchased from Biological Industries (Kibbutz Beit Haemek, Israel). We obtained O-(2-Acetamido-2-deoxy-D-glucopyranosylidene) amino-N-phenylcarbamate (PUGNAc), SB431542; d-glucosamine (GlcN), N-acetyl-d-glucosamine (GlcNAc), d-galactosamine (GalN), N-acetyl-galactosamine (GalNAc), d-mannosamine (ManN), and acetyl-d-mannosamine (ManNAc) from Sigma-Aldrich (Sigma-Aldrich, St. Louis, MO); we purchased high-capacity complementary (c) DNA kits, real-time reverse transcriptase-polymerase chain reaction (RT-PCR) kits, and probes from Applied Biosystems (Frederick, MD, USA). The mouse monoclonal immunoglobulin M (IgM) antibodies, STRO-1 and CD-146, and fluorescein isothiocyanate- (FITC-) conjugated goat anti-mouse IgM secondary antibodies were provided by Invitrogen (Carlsbad, CA, USA). The mouse monoclonal antibodies against O-linked N-acetylglucosamine (O-GlcNAc) and GnT-V were obtained from Abcam (Cambridge, UK). We purchased the rabbit polyclonal IgG primary antibodies against GnT-III and OGT from GeneTex, Inc. (Alton Pkwy, Irvine, USA). The horseradish peroxidase- (HRP-) conjugated goat anti-mouse and anti-rabbit IgG were purchased from Chemicon International (Billerica, MA, USA). We obtained the antibodies to TGF-*β*r types I and II, Smads family members from Cell Signaling Technology (Danvers, MA, USA). A set of siGENOME* SMART* pool siRNAs targeting Mgat5 mRNA came from Dharmacon (Chicago, IL, USA). We purchased nonsilencing control siRNA duplexes from Santa Cruz Biotechnology, Inc. (Delaware Avenue, CA, USA).

### 2.2. Culture and Identification of DPSCs

We cultured dental pulp cells from pulp tissue of developing third molars obtained from healthy young orthodontic patients according to detailed protocols described previously [15]. The institutional review board of our hospital approved the research protocol for this study. To sort out the stem cells with STRO-1 and CD-146 expression, we incubated cells in passage 3 with primary antibodies against STRO-1 (1 : 10) at 4°C. After washing with 2% FBS/PBS twice, cells were incubated with an FITC-conjugated secondary antibody for 30 min. Then cells were washed twice more and incubated with a CD146 antibody (1 : 50) for another 30 min. Finally, we washed the cell pellet after centrifugation and resuspended it in 500 *μ*L of a 2% FBS/PBS solution for sorting, using a FACSAria system (BD Biosciences, San Jose, CA, USA). We examined the mRNA and protein expression of GnT-V and GnT-III for the subpopulation of dental pulp stem cells with STRO-1^+^/CD146^+^ double-positive expression (denoted by DPSC) and the subpopulation of dental pulp cells with STRO-1^−^/CD146^−^ double-negative expression (denoted by DPC).

We assessed proliferation rates of DPSCs and DPCs by seeding cells with the initial density of 1 × 10^4^/cm^2^ on six-well plates, and MTT activity was measured at days 1, 3, 5, and 7. Cells' colony-forming unit fibroblast (CFU-F) assay was performed as described previously [15]. Briefly, cells were seeded at a density of 1 × 10^4^ cells onto 100 mm plates, cultured for 14 days, then fixed with 4% paraformaldehyde, and stained using 0.1% (w/V) toluidine blue. Aggregates of more than 50 cells were considered colonies.

### 2.3. Treatment of DPSCs with Hexosamine Derivatives

**Figure 1:**
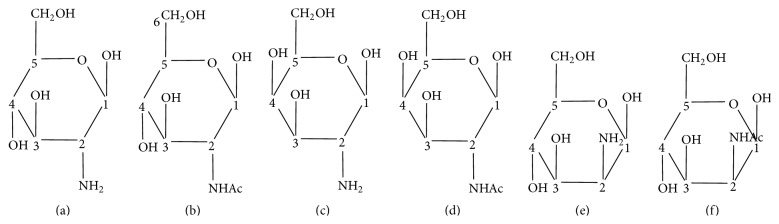
Structural formulas of hexosamines and their N-acetyl derivatives. (a) d-glucosamine (GlcN), (b) N-acetyl-d-glucosamine (GlcNAc), (c) d-galactosamine (GalN), (d) N-acetyl-galactosamine (GalNAc), (e) d-mannosamine (ManN), and (f) N-acetyl-d-mannosamine (ManNAc).

This study tested three types of hexosamines and their N-acetyl derivatives with similar structures (Figure 1). DPSCs were initially plated at a cell density of 1 × 10^4^ cells/cm^2^ and cultured to reach confluency. Then we treated the cells with various hexosamine derivatives (0.005 mg/mL) for further analysis, as described below. We prepared SB431542, an inhibitor of the TGF-*β* type I receptor, as a stock solution in dimethyl sulfoxide (DMSO) and used it at a final concentration of 10 *μ*M. PUGNAc, an inhibitor of O-GlcNAcase, was used at a final concentration of 10 *μ*M to increase the cells' level of O-GlcNAc modification.

### 2.4. RNA Isolation and Quantitative Real-Time PCR

Total cellular RNA was extracted by the methods described previously [16]. ALP, OCN, Mgat5, and Mgat3 genes were detected by quantitative real-time TaqMan RT-PCR with predesigned assays (assay ID: Hs00758162_m1, Hs01587813_g1, Hs00159136_m1, and Hs02379589_s1) using an ABI Prism 7900 Sequence Detection System (Applied Biosystems, Foster City, CA, USA). The annealing and extension temperature was set to 60°C for 40 cycles. Data were collected and analyzed with instrument spectral compensation using Applied Biosystems SDS 2.1 software. To calculate the difference in the Ct values of the target genes and control, we used the reference gene, glyceraldehyde-3-phosphate dehydrogenase (GAPDH, assay ID: Hs99999905_m1), as an internal control. Data of the experimental groups are expressed as a ratio to the control (day 0 untreated samples).

### 2.5. Assessment of Alkaline Phosphatase (ALP) and Mineralized Matrix Deposition

After treatment with various hexosamine derivatives, we evaluated the ALP activity and extracellular matrix mineralization of DPSCs by cytochemical staining as previously described [15]. Briefly, cells were fixed with 4% paraformaldehyde and stained by incubation with freshly prepared stock substrate solution for 30 min. ALP activity was measured using a spectrophotometric method based on the hydrolysis of p-nitro-phenyl phosphate into p-nitrophenol [16]. We cultured the negative control cells in regular medium and the positive control cells in mineralizing medium containing osteogenic supplements (OS) including ascorbic acid (0.05 mg/mL), *β*-glycerophosphate (10 mM), and dexamethasone (10^−7^ M).

After the DPSCs had been cultured in regular medium for the first 14 days in the presence of hexosamine, they were continuously cultured in mineralizing medium for another 14 days. We then examined mineralized matrix deposition by 2% alizarin red S (ARS) staining and used an ARS-based spectrophotometric method for quantitative assessment [17].

### 2.6. Western Blot Analysis

To extract cytoplasmic and nuclear proteins, we used ice-cold RIPA buffer (150 mM NaCl, 50 mM Tris, 1% NP-40, 1 mM sodium vanadate, 1 mM EDTA, and 0.05% sodium dodecylsulfate (SDS); pH 7.5) and buffer C (20 mM Tris-HCl at pH 7.9, 20% glycerol, 0.1 M KCl, and 0.2 mM EDTA at pH 7.9). We used electrophoresis and Western blot analysis to detect phosphorylated and total levels of Smad2 signaling molecules and total levels of Smad4 and TGF-*β*r type I. Proteins were diluted in 6x Laemmli's sample buffer, denatured at 95°C for 5 min, resolved by 12% SDS-polyacrylamide gel electrophoresis (PAGE), and electrophoretically transblotted onto polyvinylidene difluoride (PVDF) microporous membranes. We incubated the membranes with blocking solution (5% bovine serum albumin (BSA) in PBS) for one hour at room temperature and probed them with various primary antibodies (1 : 1000 dilution ratios for Smad family members, TGF-*β*r type I, GnT-V, and GnT-III) overnight at 4°C. Membranes were incubated with horseradish peroxidase- (HRP-) conjugated secondary IgG antibodies (1 : 10^4^) for one hour and checked with HRP substrates. Blots needing reprobing were stripped with stripping buffer for 5 to 10 min.

### 2.7. Small Interfering siRNA Transfection

We used a specific silencer for Mgat5 mRNA (siRNA targeted against Mgat5, siMgat5) to knockdown Mgat5 gene and Mgat5-encoded GnT-V protein expression in DPSCs. A serial working concentration (5, 50, and 100 nM) of Mgat5 siRNA was tested, with 50 nM determined as the optimal concentration.

Prior to transfection, we washed 60%–80% confluent cultures of DPSCs with PBS and incubated them in serum- and antibiotic-free medium. We prepared siRNA-Lipofectamine 2000 complexes and incubated them for six hours at 37°C. Different concentrations of siMgat5 ranging from 5 to 100 nM were tested and the optimal concentration for silencing Mgat5 was 50 nM. After an incubation period of six hours, we added an equal volume of normal medium containing two-times normal serum without removing the transfection mixture. A mock-transfection control was created by treating cells with transfection reagents without adding siRNA.

### 2.8. Statistical Analysis

Data are presented as the mean and standard deviation of three independent replicates. We checked differences between the treated group and untreated controls using Student's *t*-test. A *p* value of <0.05 was considered statistically significant.

## 3. Results

### 3.1. Expression of GnT-V and GnT-III in DPSCs and DPCs and Colony-Forming Efficiencies of siMgat5-Transfected DPSCs

**Figure 2:**
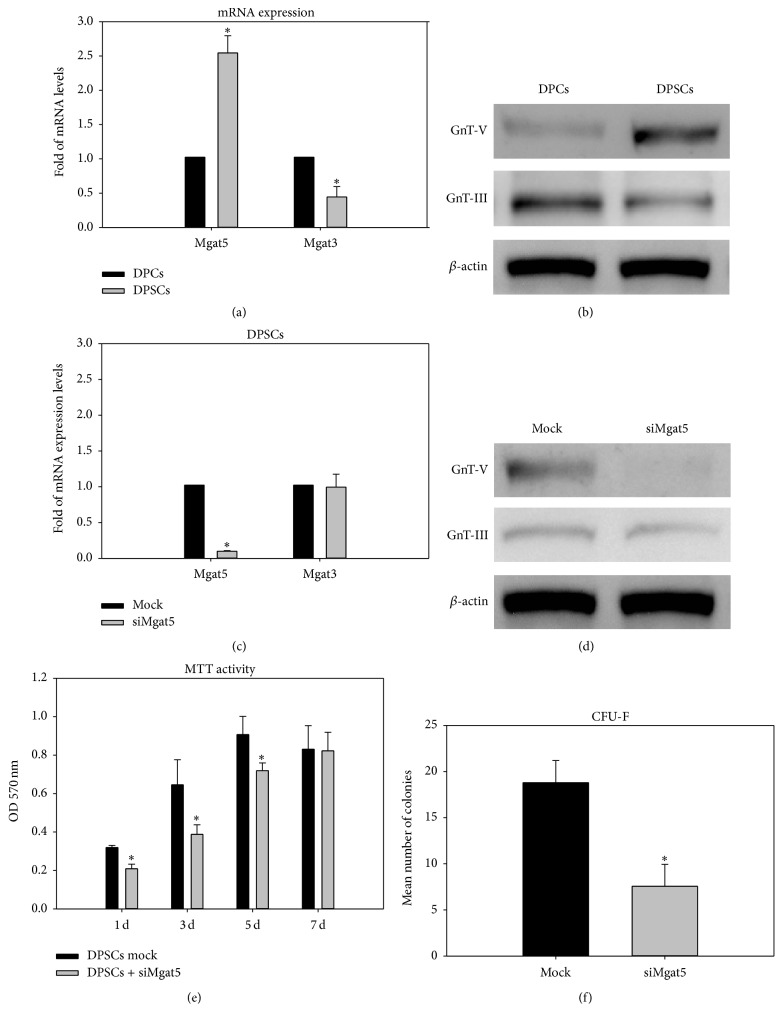
Mgat5/Mgat3 mRNA expression and GnT-V/GnT-III protein expression in dental pulp cells (DPCs) and dental pulp stem cells (DPSCs). (a) mRNA levels of Mgat5 and Mgat3 in DPSCs and DPCs, (b) Western blot image of GnT-V and GnT-III protein in DPSCs and DPCs, (c) mRNA expression of Mgat5 and Mgat3 in the DPSCs transfected with siMgat5, (d) protein expression of GnT-V and GnT-III in the DPSCs transfected with siMgat5, (e) MTT activities of siMgat5-transfected and mock control DPSCs, and (f) colony-forming unit fibroblast (CFU-F) assay of siMgat5-transfected and mock control DPSCs. Asterisks indicate a significant difference between two groups (*p* < 0.05).

Figures 2(a) and 2(b) show the higher expression of Mgat5 mRNA and GnT-V protein in DPSCs than in DPCs. Compared to the mock control, the expression of Mgat5 mRNA and GnT-V protein was substantially inhibited in the DPSCs transfected with Mgat5 siRNA (Figures 2(c) and 2(d)). Moreover, the DPSCs with GnT-V knockdown showed an obvious decrease in proliferation rate and colony-forming efficiency compared to the mock control (Figures 2(e) and 2(f)).

### 3.2. Effects of Hexosamine Derivatives on mRNA Expression of Osteogenic Genes

**Figure 3:**
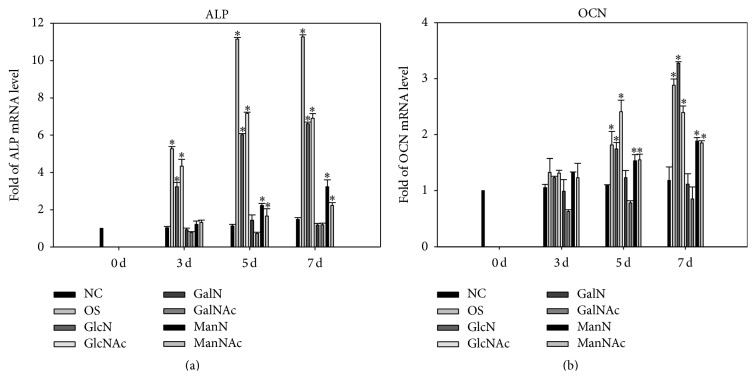
Differential regulation of GlcN/GlcNAc, GalN/GalNAc, and ManN/ManNAc on the mRNA expression of osteogenic genes in dental pulp stem cells (DPSCs). (a) Alkaline phosphatase (ALP) and (b) osteocalcin (OCN). We used cells grown in regular medium as the negative control (NC) and used those in mineralizing medium containing osteogenic supplements (OS) as the positive control. The mRNA levels were assessed by a TaqMan RT-PCR after treatment with various hexosamine derivatives (0.005 mg/mL) for 3, 5, and 7 days. Each of the targeted genes was normalized to glyceraldehyde-3-phosphate dehydrogenase (GAPDH). Results are expressed as the fold of each experimental group relative to the NC group at day 0 (mean ± SD). ^*∗*^ *p* < 0.05.

Figure 3 shows the mRNA expression of ALP and OCN genes in the DPSCs treated with various hexosamine derivatives for 3, 5, and 7 days. The cells grown in regular medium were used as the negative control (NC), and those in mineralizing medium containing osteogenic supplements (OS) were the positive control. The ALP mRNA level increased 6- to 7-fold in GlcN/GlcNAc-treated cells and 2- to 3-fold in ManN/ManNAc-treated cells on day 7 (Figure 3(a)). The OCN gene was also upregulated by treatment with GlcN/GlcNAc and ManN/ManNAc, by approximately 2- to 3-fold at later observation time points (days 5 and 7) (Figure 3(b)). These data reveal that GlcN/GlcNAc and ManN/ManNAc, but not GalN/GalNAc, upregulate the expression of osteogenic genes in DPSCs.

### 3.3. Effects of Hexosamine Derivatives on DPSC Osteogenic Differentiation and Mineralization

**Figure 4:**
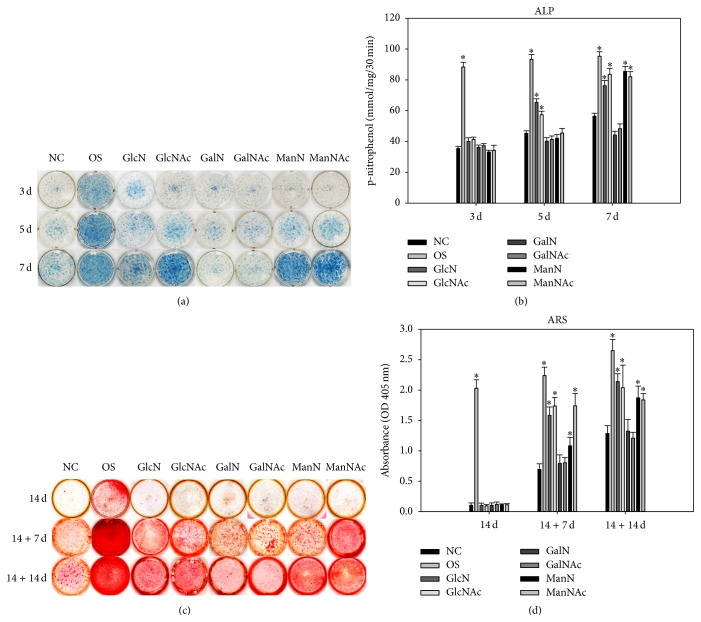
Differential effects of GlcN/GlcNAc, GalN/GalNAc, and ManN/ManNAc on alkaline phosphatase (ALP) activity and mineralized matrix deposition of dental pulp stem cells (DPSCs). (a) ALP cytochemical staining. The negative control (NC) cells were cultured in regular medium, and the positive control cells were cultured in mineralizing medium containing osteogenic supplements (OS). (b) ALP enzyme activity. (c) Alizarin red S (ARS) staining to assess mineralized matrix deposition in long-term cultures. Cells were treated with 0.005 mg/mL hexosamine derivatives for 14 days (control), followed by further culture in the presence of OS for an additional 7 or 14 days (+7 d and +14 d). (d) Quantitative assessment of ARS. Data are shown as the mean ± SD of independent triplicate cultures. For each time point, statistical significance of the difference between the experimental and the NC groups is indicated by asterisks (*p* < 0.05).

We assessed the ALP activity of DPSCs after hexosamine treatment for 3, 5, and 7 days (Figures 4(a) and 4(b)). The ALP activity of the GlcN/GlcNAc and the ManN/ManNAc groups significantly increased with the duration of the culture period. In contrast, no elevation of ALP activity was noted for the GalN/GalNAc groups. Although GlcN/GlcNAc and ManN/ManNAc triggered early osteogenic differentiation by increasing ALP activity, we detected no obvious mineralized matrix deposition in the DPSCs treated only with hexosamine derivatives for 14 days (Figures 4(c) and 4(d)). Therefore, the hexosamine-treated DPSCs were continuously cultured in mineralizing medium containing osteogenic supplements (OS) for an additional 7 and 14 days (14 + 7 d and 14 + 14 d). Subsequently, we assessed the mineralized matrix deposition using ARS staining. We noted obvious enhancement of mineralized matrix deposition in the GlcN/GlcNAc and ManN/ManNAc groups, but not in the GalN/GalNAc groups (Figures 4(c) and 4(d)).

### 3.4. Effects of Hexosamine Derivatives on O-GlcNAc Modification and TGF-*β* Signaling

**Figure 5:**
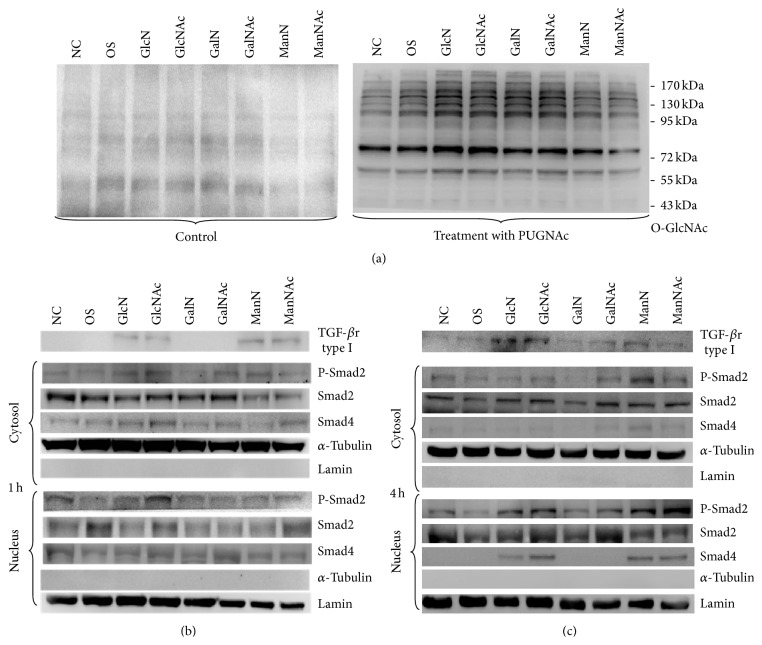
Effects of GlcN/GlcNAc, GalN/GalNAc, and ManN/ManNAc on the expression of O-GlcNAc, TGF-*β*r type I, and Smads proteins in dental pulp stem cells (DPSCs). The cells grown in regular medium were used as the negative control (NC), and those in mineralizing medium containing osteogenic supplements (OS) were used as the positive control. Expression of different O-linked glycoproteins of approximately 180, 145, 100, 63, 58, 54, and 45 kDa was detected after treatment with PUGNAc (10 *μ*M) for four hours. (a) Expression of TGF-*β*r type I (52 kDa) and phosphorylated Smad2 protein (60 kDa) in the cytosol and nuclei at 1 hr (b) and 4 hr (c).

Given that GlcN/GlcNAc, ManN/ManNAc, and GalN/GalNAc exerted variable effects on osteogenic differentiation of DPSCs, we further investigated whether they are associated with the type of glycosylation. In fact, the O-GlcNAc levels were similar among the different hexosamine-treated groups (Figure 5(a), left panel). PUGNAc is a powerful and low-toxicity inhibitor of O-GlcNAcase that is widely used to increase the cellular level of O-GlcNAc modification [16]. Thus, we used PUGNAc to check the effects of increased expression of O-linked-glycans in hexosamine-treated DPSCs. As expected, PUGNAc obviously increased the levels of O-linked glycoproteins. The presence of PUGNAc induced more O-linked glycoprotein expression in the GlcN/GlcNAc and GalN/GalNAc groups compared to the NC control group, in which cells were cultured in regular medium without hexosamine treatment (Figure 5(a), right panel). Interestingly, PUGNAc not only did not increase, but actually decreased ALP activity in the GlcN/GlcNAc and GalN/GalNAc groups compared to the NC control group (Figure 7).

Given that O-linked glycosylation may be unimportant in the hexosamine-induced osteogenic differentiation of DPSCs, this study further investigated whether the effect was associated with the N-linked glycosylated TGF-*β*r and subsequent Smads signaling. Figures 5(b) and 5(c) show that GlcN/GlcNAc and ManN/ManNAc were more effective than GalN/GalNAc in elevating the level of TGF-*β*r type I. Neither the expression of Smad2/4 nor the phosphorylation of Smad2 was affected by hexosamine treatment for one hour. However, the level of P-Smad2, especially in the nucleus, was increased by treatment with GlcN/GlcNAc and ManN/ManNAc for four hours. Smad4 was also detected at hour four in the nucleus, but not in the cytoplasm of the cells treated with GlcN/GlcNAc and ManN/ManNAc. This observation substantiates the functional role of Smad4, which interacts with regulatory Smads for translocation from cell nucleus to cytoplasm. These results suggest that GlcN/GlcNAc and ManN/ManNAc affect DPSCs by modulating the level of TGF-*β*r type I and subsequent activation of Smads signaling.

### 3.5. Effects of SB431542 and siMgat5 Transfection on TGF-*β* Signaling and ALP Activity in Hexosamine-Treated DPSCs

**Figure 6:**
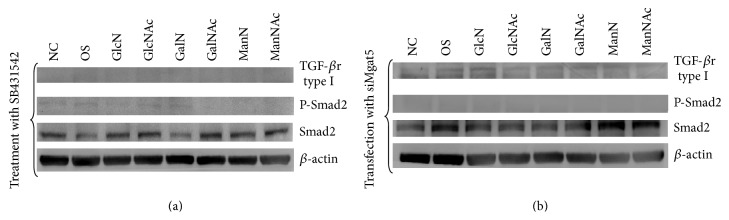
Effects of SB431542 and SiMgat5 transfection on the expression of TGF-*β*r type I and Smad2 phosphorylation in DPSCs treated with GlcN/GlcNAc, GalN/GalNAc, and ManN/ManNAc for four hours. We used cells grown in regular medium as the negative control (NC) and those in mineralizing medium containing osteogenic supplements (OS) as the positive control. (a) SB431542 abolished TGF-*β*r type I expression and Smad2 phosphorylation; (b) transfection with siMgat5 to knockdown GnT-V also abolished TGF-*β*r type I expression and Smad2 phosphorylation.

**Figure 7:**
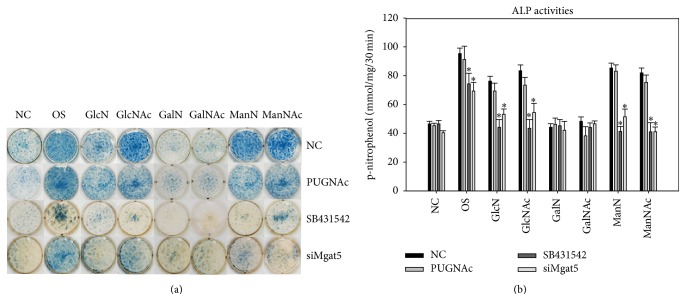
Differential effects of increased O-linked glycosylation, inhibition of TGF-*β*r type I, and GnT-V knockdown on alkaline phosphatase (ALP) activity in DPSCs treated with GlcN/GlcNAc, GalN/GalNAc, and ManN/ManNAc for 7 days. We used the cells grown in regular medium as the negative control (NC) and those in mineralizing medium containing osteogenic supplements (OS) as the positive control. (a) ALP cytochemical staining in hexosamine-treated DPSCs by treatment with PUGNAc, SB431542, or siMgat5 transfection. (b) ALP enzyme activity. Representative data are shown as the mean ± SD of independent triplicate cultures. Asterisks indicate a significant difference between the experimental and the NC groups (*p* < 0.05).

DPSCs were cultured in regular medium and treated with SB431542 for four hours concomitantly with various hexosamine derivatives. Surprisingly, the hexosamine-enhanced expression of TGF-*β*r type I and phosphorylated-Smad2 was completely abolished in response to SB431542 (Figure 6(a)). SB431542 also inhibited ALP activity in the GlcN/GlcNAc and ManN/ManNAc groups (Figure 7). However, treatment with GalN/GalNAc did not increase the ALP activity of DPSCs, regardless of the presence or absence of SB431542.

Figure 6(b) shows that siMgat5 transfection of DPSCs significantly inhibited the hexosamine-induced TGF-*β*r type I expression and Smad2 phosphorylation. A dramatic decrease of ALP activity was also noted for the GlcN/GlcNAc and ManN/ManNAc groups in comparison to the NC control group (Figure 7). As described previously, the expression of GnT-V protein was almost completely abolished in the siMgat5-transfected DPSCs (Figures 2(c) and 2(d)). These results indicate that GnT-V is involved in the hexosamine-induced osteogenic differentiation of DPSCs.

## 4. Discussion

To our knowledge, this is the first report showing the expression of GnT-V in DPSCs and the variable effects of hexosamine derivatives on osteogenic differentiation in DPSCs. Our results reveal that GlcN/GlcNAc and ManN/ManNAc, but not GalN/GalNAc, trigger the early osteogenic differentiation of DPSCs in the absence of osteogenic supplements by modulating the level of TGF-*β*r type I and Smad2 signaling. In the presence of osteogenic supplements, long-term cultures of GlcN/GlcNAc or ManN/ManNAc-treated DPSCs showed an increased mineralized matrix deposition, a late-stage marker of osteogenic differentiation. As shown in Figure 1, the C-4 hydroxyl group of GalN/GalNAc is in the opposite direction to that of GlcN/GlcNAc and ManN/ManNAc. In other words, GalN is the epimers of GlcN and ManN on carbon C-4. Moreover, GlcN and ManN are epimers at the C-2 position. This suggests that the biological functions of C-2 epimers of hexosamines and their N-acetyl derivatives are similar, regardless of the presence or absence of an acetyl group. This implies that the biological effects of hexosamine derivatives on DPSCs depend in a sensitive manner on the stereoisomerism instead of the presence of an acetyl group.

GnT-V modifies many glycoproteins on the cell surface, for example, the integrin family or growth factor receptors with high or low numbers of N-glycans [18, 19]. N-linked glycosylation has been shown to play a crucial role in cell surface transportation of TGF-*β*r type II and ligand binding of TGF-*β*r type I [20, 21]. The results of this study suggest that GnT-V catalyzed N-glycan-branching is important for the regulation of TGF-*β* signaling in the hexosamine-induced osteogenic differentiation of DPSCs. UDP-GlcNAc is formed via the hexosamine pathway and used as a sugar donor in the biosynthesis of N-glycan branching glycoproteins [11, 22]. The availability of exogenous hexosamines may regulate the production of UDP-GlcNAc and affect the level of cell surface glycoproteins.

GnT-V catalyzes the addition of *β*1,6-GlcNAc to branched* N*-linked glycoproteins; then the N-glycans are extended with GalNAc and ManN and capped with sialic acid or fucose to serve as ligand receptors including EGFR, CTLA-4, GLUT4, and TGF-*β*r [6, 8]. Some malignant phenotypes have been reported to be associated with N-glycans branching catalyzed by GnT-V [23], whereas GnT-III has an antagonistic role in GnT-V and contributes to the suppression of cancer metastasis [24]. It was recently reported that GnT-V and its reaction products were distinctly expressed in proliferating neural/progenitor cells, whereas its expression was diminished in differentiated cells [25]. Our study reveals a significant decrease of colony-forming efficiency in the DPSCs transfected with a specific small interfering RNA to silence the Mgat5 gene (Figure 2(f)), implying that the expression of GnT-V is involved in the regulation of stem cell behaviors. Our previous work found that DPSCs (STRO-1^+^/CD146^+^ cells) are more responsive to exogenous GlcN than DPCs (STRO-1^−^/CD146^−^ cells) in the upregulation of osteogenic genes [15]. Our study further shows that the DPSCs exhibited higher expression of GnT-V and lower expression of GnT-III compared to the DPCs at both the transcriptional and translational levels (Figures 2(a) and 2(b)). The exogenous GlcN/GlcNAc and GalN/GalNAc enhanced ALP activity of DPSCs in the absence of osteogenic supplements, whereas the DPSCs with GnT-V knockdown significantly lost inducibility of ALP expression (Figure 7(a)).

To investigate if O-linked glycosylation plays a role in the effects of hexosamine derivatives on DPSCs, we used a low-toxicity powerful inhibitor of O-GlcNAcase, PUGNAc, to increase the level of O-glycosylation modification [26]. Interestingly, the presence of PUGNAc, GlcN/GlcNAc, and GalN/GalNAc increased the O-glycosylation modification but mildly decreased the ALP activity in DPSCs (Figures 5(a) and 7(a)). These results suggest that the involvement of O-linked glycosylation may be not as important as N-linked glycosylation in hexosamine-induced osteogenic differentiation of DPSCs.

TGF-*β*r type I is a key regulator determining the specificity of intracellular signals for osteogenic differentiation [27, 28]. Generally, TGF-*β*r type I is phosphorylated by the constitutively active type II receptor kinase in TGF-*β*-mediated responses [29]. Constitutively active TGF-*β*r type I has been reported to exert TGF-*β* signals in the absence of ligand or functional TGF-*β*r type II [30]. SB431542 is a specific inhibitor of TGF-*β*r type I and TGF-*β* signaling [31]. In our study, GnT-V knockdown or treatment with SB431542 significantly abolished TGF-*β*r type I and subsequent Smad2 phosphorylation, as well as ALP activity, in the DPSCs treated with GlcN/GlcNAc and GalN/GalNAc (Figures 6(a) and 7(a)). Again, these results suggest that TGF-*β*r type I plays a critical role in the biological effects of hexosamine derivatives. The process of endocytosis may influence receptor density at the cell surface. It has previously been reported that Mgat5-modified N-glycans on TGF-*β* receptors at cell-surface cross-linked Gal-3; thus their removal by constitutive endocytosis was delayed [32]. Further studies are required to investigate if exogenous GlcN/GlcNAc and ManN/ManNAc modify the balance between surface retention of TGF-*β*r type I against its loss via endocytosis.

In conclusion, GlcN/GlcNAc and ManN/ManNAc promote the osteogenic differentiation of DPSCs mainly via modulating the level of N-glycan-branching TGF-*β*r type I. The subsequent phosphorylation of Smad2 signaling molecules further increased ALP activity and enhanced extracellular matrix mineralization. These stimulatory effects were significantly inhibited either by treatment with SB431542 or transfection of Mgat5 mRNA silencer. Further efforts are required to investigate the effects of GlcN/GlcNAc and ManN/ManNAc in vivo for their application in dentinogenic/osteogenic tissue engineering.
